# Can Faecal Eosinophil Cationic Protein and β-Defensin-2 Levels Be Useful in the Diagnosis and Follow-Up of Infants with Milk-Protein-Induced Allergic Proctocolitis?

**DOI:** 10.3390/nu17172796

**Published:** 2025-08-28

**Authors:** Grażyna Czaja-Bulsa, Monika Łokieć, Arleta Drozd

**Affiliations:** 1 Chair and Department of Paediatrics and Paediatric Nursing, Pomeranian Medical University, 70-204 Szczecin, Poland; 2 Clinical Department of Paediatrics University Hospital, 65-046 Zielona Góra, Poland; monika.taczalska@op.pl; 3 Department of Applied Microbiology and Human Nutrition Physiology, Faculty of Food Sciences and Fisheries, West Pomeranian University of Technology, ul. Papieża Pawła VI 3, 71-459 Szczecin, Poland; arleta.drozd@gmail.com

**Keywords:** cow’s milk allergy, non-IgE mediated allergy, food-protein-induced allergic proctocolitis, eosinophil cationic protein, β-defensins, children

## Abstract

Objective: The aim of our study was to investigate whether faecal concentrations of eosinophil cationic protein (fECP) and human β-defensins (HBD2s) are significantly elevated in children with cow’s milk-protein-induced allergic colitis (MPIAP) and whether a monthly milk-free diet reduces these markers. Materials and methods: This was a single-centre, prospective, observational cohort study involving 70 infants with MPIAP, aged 1–3 months, and 30 healthy controls of the same age. The concentrations of fECP and HBD2 were measured using the ELISA method (IDK^®^ Eosinophil Cationic Protein and β-Defensins ELISA Kit, Immunodiagnostik AG, Germany). Diagnosis of MPIAP was confirmed with an open milk challenge test. Results: The concentrations of fECP and HBD2 proved useful in evaluating MPIAP treatment with a milk-free diet, where the resolution of allergy symptoms and a significant (*p* = 0.0000) decrease in the concentrations of both biomarkers were observed after 4 weeks of following the diet. The concentrations of fECP and HBD2 were still higher than those in the control group. High concentrations of fECP can be helpful in diagnosing MPIAP (100% sensitivity), but the low specificity of the assay means that there is a risk of diagnosing MPIAP in one in six children who do not have the disease. The concentrations of HBD2 have low sensitivity, so one in four children with MPIAP will not be confirmed to have the disease using this indicator. Conclusions: fECP and HBD2 can be used to monitor the resolution of colitis in infants with MPIAP treated with a milk diet, indicating a slower resolution of allergic inflammation than the resolution of allergic symptoms. Therefore, neither of the parameters are useful for the diagnosis of MPIAP.

## 1. Introduction

Cow’s milk protein allergy (CMPA) is common in early infancy. According to various review papers, its prevalence during early infancy is 1–4%, rising to 10% when the diagnosis is based on clinical suspicion or caregiver assessment. The incidence of CMPA is less than 1% when verified by an oral provocation test (OFC) [1,2,3,4,5]. A large systematic review and meta-analysis of allergy prevalence in Europe for eight major foods in all age groups was recently published. The prevalence of CMPA was shown to be 5.7%, but after verification with the OFC test, it declined to 0.3% [6].

There are three types of CMPA: IgE-dependent (IgE-CMPA), IgE-independent (non-IgE-CMPA), or mixed IgE/non-IgE-mediated (mixed CMPA) [7,8,9,10,11,12,13,14,15]. When CMPA is diagnosed, milk should be eliminated from the diet, resulting in the resolution of symptoms. This is followed by a milk provocation test, which results in the recurrence of pre-existing symptoms, so parents are often reluctant to perform it, making the diagnosis difficult.

In IgE-CMPA, in which serious life-threatening symptoms can occur, provocation tests should be performed in the hospital. In non-IgE-CMPA, dangerous symptoms, in the form of vomiting with dehydration, occur in only one form: food-protein-induced enteritis syndrome (FPIES). In this case, food provocations are performed in the hospital. In patients with other forms of non-IgE-CMPA, provocations are performed under supervision in an outpatient clinic. Patients often present with various gastrointestinal symptoms and skin lesions in the form of atopic dermatitis [5,7,8,9,10,13,14].

In IgE-CMPA, to confirm milk sensitisation, skin tests with milk can be performed or specific IgE can be determined for milk and its allergens. In non-IgE-CMPA, no marker is known to confirm milk sensitisation. In recent years, there has been increasing interest in finding such a factor, preferably one that is non-invasive, highly sensitive, widely available, simple to perform, and easy to use in infants. Such factors could be biomarkers found in faeces [16].

In non-IgE-CMPA, the most commonly tested biomarker is faecal calprotectin (FC). Some studies are looking at other faecal biomarkers, such as tumour necrosis factor α (TNF-α), human β-defensins (HBD2), eosinophil cationic protein (fECP), eosinophil-derived neurotoxin (fEDN), and zonulin-related protein (FZRP).

Calpoprotectin (FC) is an indicator of neutrophilic inflammation. During intestinal inflammation, FC is released from neutrophils attacking tissue. Large amounts of it are found in the intestinal lumen and are excreted in the stool. In recent years, FC has been recognised and validated as a biomarker of intestinal inflammation in children and adults with inflammatory bowel disease (IBD) [17,18]; it has also been used to differentiate irritable bowel syndrome from IBD. In other types of intestine and colitis inflammation, FC has not been thoroughly studied [19]. The first study indicating its usefulness in the diagnosis of CMPA was published in 2011 [20].

Unfortunately, subsequent studies have shown high variability in FC concentrations in stool in both healthy children and children with CMPA [21,22,23,24,25,26]. Furthermore, significant differences between researchers were found, which excludes the usefulness of FC in the diagnosis of CMPA. Individual analysis of FC concentrations in the stool of each child showed a significant increase in FC in young infants with CMPA and a decrease after only 4 weeks of a milk-free diet [27,28,29,30,31].

Calprotectin is therefore useful for assessing the resolution of allergic inflammation when following a milk-free diet. Stool samples collected on the second day of life from 70 newborns showed higher levels of FC in neonates delivered via Caesarean section. This finding may indicate low-grade inflammation in response to dysbiosis.

FZRP is a marker of increased intestinal permeability [32,33,34,35,36]. FZRP has been found to be slightly variable in healthy children, increasing in children with CMPA and decreasing in those following a milk-free diet [27]. Unfortunately, one-fifth of children do not synthesise FZRP, which is an important limitation in the diagnosis of non-IgE CMPA [21,34]. FZRP is a better parameter for diagnosing CMPA than FC, but it has only been evaluated in one study [27]; this should be assessed in further studies.

TNF-α is a pro-inflammatory cytokine produced by monocytes and macrophages. It is involved in the pathophysiology of intestinal allergy by initiating this process of increased intestinal permeability and mucosal infiltration by leukocytes [37]. In the case of allergic disorders, Majamaa et al. showed that particularly high concentrations of TNF-α were found in patients manifesting delayed-onset allergy reactions [38]. This relationship was not confirmed by Rycyk et al. [39].

EDN levels are a marker of intestinal inflammation mediated by eosinophils, suggesting eosinophil activation and degranulation in the gut. Eosinophils contain cytotoxic granules comprising multiple antimicrobial proteins, including ECP and EDN, involved in host defence against viral and parasite invasion. High levels of EDN have been described in active ulcerative colitis, Crohn’s disease, and in young children with non-IgE-CMPA [39,40,41,42]. Reference values for EDN levels in children have not been established. There is a high intrapersonal variability in EDN levels in children aged 0 to 4 years [40]. In 27 newborns with MPIAP, there was an increase in faecal EDN when undergoing the oral challenge test with milk; this was significantly higher than in those with gastrointestinal functional disorders [29].

ECP is a protein found in eosinophil granules, which are released after activation in intestinal inflammation. fECP is considered to be a specific indicator of eosinophilic inflammation. Several studies have evaluated fECP in CMPA, but they have not produced consistent results.

HBD2 defensins are also activated in intestinal inflammation, but they have only been studied in one study involving children with non-IgE-CMPA.

The purpose of our study was to evaluate the usefulness of two faecal biomarkers, fECP and HBD2, in children with milk-protein-induced allergic proctitis (MPIAP) in the diagnosis and assessment of remission of allergic inflammation after 1 month of following a milk-free diet.

## 2. Materials and Methods

This was a single-centre prospective observational cohort study involving 70 children with MPIAP, with a mean age of 2 months (1–3 months), and 30 healthy infants. The children had been diagnosed with suspected gastroesophageal reflux disease (GERD), although a link between their symptoms and milk allergy was ruled out.

The study and control groups did not differ in gender, age, height and weight, feeding regimen at the time of diagnosis, or family history of allergic diseases. Eosinophilia and anxiety were more common in children with MIAP, while anaemia was more common in children in the control group who had GERD. Detailed data are presented in Table 1.

This study was conducted for 2 years (2020–2022). The children were patients of the Pediatric Gastroenterology and Allergology Clinic in Szczecin. All children had symptoms indicative of MPIAP, such as diarrhoea with blood or streaks of blood. One in four children also suffered from atopic dermatitis (AD).

This study enrolled 96 children with diarrhoea with blood or blood strands and occasionally atopic dermatitis. After preliminary tests, 16 children were diagnosed with constipation, gastrointestinal infections, intussusception, anal fissures, and coagulation disorders. In 80 infants, MPIAP was suspected, and a milk-free diet was initiated for 4 weeks. In total, 76 of the children improved; in the 4 in whom the milk-free diet did not induce an improvement, their diagnosis was widened as we performed gastroduodenoscopy, colonoscopy, or scintigraphy for Meckel’s diverticulum.

In the 76 infants who significantly improved while following a milk-free diet, an open oral food challenge test (OFC) with milk was performed. The OFC test was positive in 70 infants who were included in the study group (MPIAP_0_ group). The children from the study group who had been following a milk-free diet for 1 month were classified as MPIAP_1_.

During the diagnostic elimination diet, a milk-free diet was initiated, comprising either milk from mothers who followed a milk-free diet, mixed feeding (breast and extensively hydrolysed cow’s milk formula, eHF), or eHF (Table 1). All of the infants studied were in a good general condition, with normal height and weight (10–97c). All children were born on time.

The first stool sample for the determination of fECP and HBD2 was taken immediately before the introduction of the milk-free diet (fECP_0_, HBD2_0_), and the second was taken after 4 weeks of following the milk-free diet (fECP_1_, HBD2_1_).

Each patient underwent a physical examination and had their medical and allergy history recorded. The criteria for including a child in the study were the presence of MPIAP, cow’s milk-only dependent symptoms, age (up to 4 months), and no coexisting chronic diseases (except atopic dermatitis as a form of CMPA). Each patient had written consent from their parents/legal guardians to participate in the controlled study and to collect and publish the results obtained. The exclusion criteria included other forms of CMPA, coexisting chronic diseases, and age over 4 months. The child was also excluded from the study if there was no written consent from parents/legal guardians for the child’s participation in the controlled study.

The age of MPIAP diagnosis denoted the age when a diagnostic milk-free diet was introduced. The diagnosis of MPIAP (FPIAP) was given according to the recommendations of the WAO and EAACI [43,44].

This research was approved on 27 January 2020 by the Pomeranian Medical University Bioethics Committee No KB-0012/5/20. Title: “Selected markers of intestinal mucosal inflammation in the diagnosis of cow’s milk protein allergy in infants and young children”.

This research was financed by statutory activities (WNoZ-319-01/s/12/2020-2022). The presented results are part of the ongoing project.

### 2.1. OFC Procedures

OFC procedures were always initiated in an outpatient setting. All children were under the supervision of a nurse and/or physician, in a room with antishock medication [45]. First, a lip test was performed (a drop of milk on the lip). After a negative lip test result, gradually increasing doses of milk were administered every 15 min, starting with 1 mL and increasing to 100 mL. Patients remained under observation for at least 2 h (usually 4–6 h) since the end of the OFC test [45,46,47]. The provocation continued at home by the child’s parents for 6 days. Each day, they gave their child a milk mixture corresponding to the volume of one meal (up to 120 mL). They recorded any adverse symptoms in the observation card. If adverse symptoms occurred, the provocation was discontinued. If no symptoms occurred within 6 days, the doctor diagnosed milk tolerance.

A positive OFC test result was found in 70 children and this was the basis for the diagnosis of CMPA. A negative provocation test was found in six children and this ruled out the presence of CMPA. All infants developed symptoms after 8 h to 3 days after milk consumption. Infants with delayed reactions during the OFC test were diagnosed with non-IgE CMPA, and those who presented symptoms were diagnosed with MPIAP.

### 2.2. Faecal Samples

The first stool sample was taken immediately before the introduction of the milk-free diet (fECP_0_, HBD2_0_) and the second one was taken after 4 weeks of following the diet (fECP_1_, HBD2_1_). Fresh stool for testing was stored for 2 days at room temperature of 15–30 degrees; after that, the samples were frozen and stored at −80 °C. All patients provided a stool specimen the day before their visit to the outpatient clinic. In the laboratory, the raw stool sample was thawed. In the case of particularly heterogeneous samples, mechanical homogenisation was used. A test strip with incisions was placed in the stool sample to completely cover it with stool. When the strip was removed, excess stool was removed, leaving 15 mg of sample for dilution. When determining ECP, the supernatant was diluted with washing buffer at a ratio of 1:4. A final dilution of 1:400 was obtained. When determining HBD2, the supernatant was diluted in washing buffer at a ratio of 1:2. A final dilution of 1:200 was obtained. For the analysis, 100 μL of the dilution was pipetted into each well.

The fECP was determined using the ELISA method (IDK^®^ ECP ELISA Kit K6811, Immunodiagnostik AG, 64625 Bensheim, Germany). The HBD2 was also assessed using the EELISA method (IDK^®^ B-Defensin ELISA Kit K6500.20, Immunodiagnostik AG, 64625 Bensheim, Germany).

Olafsdottir et al. found that stool collected from a nappy had a 30% higher FC concentration due to water absorption [48]. In all infants, stools were collected in the same way (from nappies) for the determination of fECP and HBD2.

### 2.3. Statistical Analysis

All data were collected in electronic form in an MS Excel spreadsheet and subject to statistical analysis. Discontinuous variables are described by number and frequency of occurrence. Continuous variables are described by median, minimum, and maximum values. Pearson’s χ^2^ test or Fisher’s exact test and Spearman’s rank correlation were used to test the statistical relationships between the discontinuous variables.

Probability (p) was calculated using two tests: the Mann–Whitney U-test for variables, for which the normality of the distributions was not satisfied, and Student’s T-test for variables with a normal distribution. When searching for the fECP and HBD2 concentration that differentiates non-diagnosed children (control group) from children diagnosed with MPIAP the best (MPIAP_0_ group), ROC curve analysis was used. The results were described by the area under the curve (AUC) and the standard error of the AUC (SE). The 95% confidence interval for the AUC (95% CI), the p-likelihood, and the coordinates of the ROC curves, i.e., the specificity and sensitivity of the study group relative to the control group, were estimated for each range of values of the continuous variable.

## 3. Results

### 3.1. Faecal Eosinophil Cationic Protein (fECP)

The median fECP concentration in the children in the control group was 0.22 mg/L (0.11–1.47 mg/L) (Table 2). The concentration was significantly lower (*p* < 0.0001) than that in the children in the study group at the time of MPIAP diagnosis, 2.36 mg/L (1.03–5.46 mg/L), and after 1 month of following a milk-free diet, 0.47 mg/L (0.14–2.58 mg/L) (*p* < 0.0001). After 1 month of following a milk-free diet, the resolution of MPIAP and AD symptoms was observed in all children. At the same time, a significant decrease in the fECP concentration was observed in all children (*p* < 0.0001). However, it did not reach the level found in the children in the control group, although it was significantly close to it; it was still significantly higher (*p* < 0.0001).

ROC curve analysis was used to find the fECP concentration that best differentiated healthy control children from those with MPIAP_0_. A concentration of 1.005 mg/L with a sensitivity of 100% and a specificity of 83% differentiated these groups of children (Table 3). All children with MPIAP_0_ reached values equal to or higher than 1.005 mg/L. Unfortunately, up to 16.7% of the control group had such high concentrations. After 1 month of treatment with a milk-free diet, fECP concentrations above 1.005 mg/L still persisted in the 12.3% children with MPIAP (87.7%).

### 3.2. Faecal β-Defensins (HBD2s)

The median HBD2 concentration in children in the control group was 3.10 ng/L (1.10–10.30 ng/L) (Table 2). It was significantly lower (*p* < 0.0001) than in children in the study group at the time of MPIAP diagnosis, 58.35 ng/L (23.70–603.20 ng/L), and after 1 month of following a milk-free diet, 9.10 ng/L (1.90–384.90 mg/L) (*p* < 0.0001). After 1 month of following a milk-free diet, the resolution of MPIAP and AD symptoms was observed in all children. At the same time, a significant decrease in the HBD2 concentration was observed in all children (*p* < 0.0001). However, it did not reach the level found in the children in the control group, although it was significantly close to it; it was still significantly higher (*p* < 0.0001).

ROC curve analysis was used to find the HBD2 concentration that best differentiated the children from the control group from those with MPIAP_0_. A concentration of 19.35 ng/L with a sensitivity of 73% and a specificity of 100% differentiated these groups of children (Table 3). Unfortunately, only 72.9% of children with MPIAP_0_ reached values equal to or higher than 19.35 ng/L. All children in the control group have lower values. After 1 month of following a milk-free diet, HBD2 concentrations above 1.005 mg/L still persisted in 28.9% of the children with MPIAP.

## 4. Discussion

Food-protein-induced allergic proctocolitis (FPIAP) is one of the most common forms of non-IgE-CMPA [14]. It was previously referred to as allergic or eosinophilic colitis [49]. It involves inflammation of the distal part of the colon following an allergic reaction to one or more foods, most commonly milk—MPIAP [50,51]. The main symptoms of MPIAP are rectal bleeding, mild diarrhoea, and the child being significantly irritated [14]. The disease usually manifests itself in the first weeks of life. In the group of children studied, symptoms appeared between 4 and 9 weeks of age, most commonly at 6 weeks. MPIAP can persist for up to 3 years, but in most children, it resolves within 12 months of life [14,52]. Research by Lozinsky et al. shows that MPIAP persisted in only 21% of children after 1 year of age [53]. Since MPIAP symptoms may resolve spontaneously without the introduction of a milk-free diet, some researchers wait 2–4 weeks before introducing the diet after diagnosis [54]. In the presented study, we did not delay the introduction of a milk-free diet, but the time from the onset of symptoms to the start of the diet ranged from 3 to 4 weeks.

ECP is a single-chain protein with a molecular weight ranging from 16 to 22 kDa [55]. The gene that codes for ECP has been located on chromosome 14q11.2; three polymorphisms have been identified [56]. ECP is one of the most important proteins of eosinophil granulocytes; it is present only in the matrix [57]. Mature eosinophil granulocytes contain 13.5 mcg ECP/10^6^ cells [58]. Unstimulated eosinophils do not produce ECP, but they can take it up from the environment. Activated eosinophils secrete ECP following the action of antibodies (IgG, IgA) or C3 and C5 complement components. During the release of ECP molecules from eosinophils, an enzymatic deglycosylation process occurs, in which inactive ECP with a high molecular weight is converted into an active variant with a low molecular weight [59]. ECP exhibits numerous biological activities, both cytotoxic and non-toxic. ECP, through its cytotoxic action, plays an important role in the host’s immune defence. The cytotoxic action of ECP is directed against viruses, parasites, and Gram-negative and Gram-positive bacteria [60]. Since ECP is secreted only by activated eosinophils, it is considered a more specific indicator of eosinophilic inflammation than the number of eosinophils in the peripheral blood. Depending on the location of allergic inflammation, ECP is present in numerous body fluids such as serum, plasma, sputum, saliva, nasal lavage, bronchoalveolar lavage (BAL), tears, synovial fluid, and faeces. Recently, fECP has been investigated as a new potential biomarker for IBD, eosinophil gastroenteritis, and colitis [61,62]. It has been demonstrated that in healthy children, the fECP concentration does not depend on the serum ECP concentration or peripheral blood eosinophil count [63]. In healthy individuals, the mean and median ECP values in faeces are 1.93 and 1.20 mcg/g, while the serum values are 13.50 and 9.54 mcg/l. Therefore, fECP only indicates the severity of eosinophil activation in the intestine and is therefore a good intestinal biomarker. The concentration of fECP does not depend on age or gender.

In our control group infants, aged 1–3 months, the median ECP in their stool was 0.22 mg/L (range: 0.11–1.47 mg/L). Most healthy children (83%) had fECP concentrations below 1.005 mg/L.

There are only a few studies evaluating the usefulness of fECP for the diagnosis of CMPA. Their results are inconclusive. Majamaa et al. found an increase in fECP during positive milk challenge in children with IgE-CMPA [38]. Contrary to these results, Saarinen et al. reported a significant increase in fECP during a positive milk challenge in 208 infants aged 7 months with non-IgE-CMPA [64]. Baldsssarre et al., characterising various intestinal markers in CMPA, recognised fECP as a marker of immediate reactions during milk challenge [65]. They noted that the increase in fECP during provocation is slow and may take several hours or even several days. It is necessary to determine the time at which this parameter should be tested [66].

In 1996, Majamaa et al. demonstrated that food allergy causes intestine allergic inflammation in children with atopic dermatitis [38]. In children with a positive milk challenge test, the concentration of ECP in faeces increases particularly in patients with immediate reactions after exposure to cow’s milk, while the concentration of TNF-alpha in faeces increases in patients with delayed reactions, confirming the different pathogenesis (with and without IgE involvement) of these two types of reactions.

Saarinen et al. studied 208 infants with CMPA at 7 months of age [64]. They found that regardless of the symptoms present, milk always caused intestinal inflammation. After 3 weeks of a milk-free diet, the fECP concentrations were higher in infants with non-IgE-CMPA compared with infants with IgE-CMPA. The highest fECP values were found in children with intestinal symptoms. After 4 weeks of milk provocation, children with non-IgE-CMPA had higher fECP concentrations than children with IgE-CMPA. These results indicate that children with CMPA without IgE have increased eosinophil activation in the intestines, i.e., increased allergic inflammation activity compared with children with IgE-CMPA.

In the present study, we evaluated fECP values in children with MPIAP, the most common form of non-IgE-CMPA. Some of them also had AD. The concentration of fECP appears to be a good intestinal marker. In the control group, the values do not show significant individual variability. In children with MPIAP, the fECP values increase significantly, which allows for the determination of a value that differentiates patients from the control group. This value is ≥1.005 mg/L. Unfortunately, as many as 17% of children in the control group have concentrations higher than this value, resulting in a risk of diagnosing MPIAP in one in six children who do not have the disease. This excludes the use of fECP in the diagnosis of MPIAP. Faecal ECP can be used to monitor the resolution of colitis in infants with MPIAP treated with a milk diet, indicating a slower resolution of allergic inflammation than the resolution of allergic symptoms.

Children with haematochezia always have higher ECP and EDN levels than children with other types of gastrointestinal allergies, indicating that this is eosinophilic colitis [49,54,65].

Calprotectin and eosinophil-derived proteins such as ECP and EDN are elevated in the faeces of children with FPIAP, indicating the simultaneous involvement of neutrophils and eosinophils in this inflammation [49,50,51,52,53,54,55,56,57]. The baseline levels of these markers are variable, but after allergen stimulation, a significant increase was observed in all patients 24 h after exposure [66,67,68]. Faecal EDN and FC markers are useful in distinguishing children with FPIAP from children with inflammatory bowel disease (IBD) and children with functional gastrointestinal disorders, in whom these markers are the same as those in children without any of these diseases [16,17,18,19,29,69].

Defensins are small, 29 to 49 amino acid cationic peptides with a molecular weight of 3–5 kDa. In humans, there are two groups: alpha and beta defensins. Defensins α are constantly present on mucosal surfaces, while defensins HBD-2, -3, and -4 are activated in response to external stimuli at sites of infection or inflammation [70]. In a healthy colon, they are expressed in very small amounts. They are broad-spectrum immunostimulators participating in innate and adaptative immunity in response to infectious diseases to maintain the balance between pathogens and normal flora [71,72]. Defensins are called endogenous antibiotics because of their broad activity against Gram-negative and Gram-positive bacteria, fungi, viruses, and protozoa [73]. Defensins disrupt microbial cell membranes, leading to the death of virulent pathogens. Defensins also participate in regulating the number and composition of commensal microbiota and tolerance to normal flora [71]. Maintaining a balance between combating pathogenic bacteria and protecting and supporting commensal microflora is crucial for maintaining homeostasis [71].

An increase in HBD2 secretion also occurs after the administration of probiotics [74].

It has been found that HBD2 is always present in the stool of premature babies and full-term newborns already in the first weeks of life [72]. It has also been found in meconium. Since HBD2 is present in stool from the first days of life, it is believed to be a component of the gut barrier against infections [75]. After 2 weeks of life, the concentration of HBD2 is the same in premature babies and full-term newborns. It has been shown that in healthy infants, the mode of delivery and feeding do not affect the HBD2 concentration [76]. When a decrease in HBD2 production is observed in premature infants, it is associated with a change in the composition of the colonic microbiota, which determines colonic inflammation and is responsible for the high risk of obtaining NEC [75]. HBD2 increases in infants with NEC.

HBD2 is involved in the first line of defence against infections by promoting interactions between innate and adaptive immunity in newborns [73]. Similarly, in children and adults with inflamed mucosa, an increased expression of HBD2 is observed, which decreases in mucosa without inflammation [77]. The mucosal expression of HBD2 increases in patients with ulcerative colitis. In contrast, it is reduced in the colonic mucosa of patients with Crohn’s disease [78]. This phenomenon has been observed in patients from Europe and America, but opposite results were obtained by researchers from New Zealand. A decrease in the defensin concentration in Crohn’s disease leads to a reduction in antibacterial killing by the mucosa [79].

The expression of defensin is altered in atopic dermatitis and allergic rhinitis [73].

Merras-Salmio et al. studied faecal HBD2 in 18 children with non-IgE-CMPA and 22 children from a control group aged 2.4–40.8 months [65]. In this study, children with CMPA presented various forms of non-IgE-CMPA. Merras-Salmio et al. found high individual variability within groups and a trend towards higher values in children with non-IgE-CMPA, but these differences were not statistically significant. In our study, we also found high levels of within-group variation. The concentrations of HBD2 were significantly higher in infants with MPIAP compared to those in children without MPIAP. In the children from Merras-Salmio et al.’s control group, higher concentrations of faecal HBD2 were obtained compared to those found in our study: 8.6–50.0 ng/mL (median 20.8 ng/mL) vs. 1.10–10.30 ng/mL (median 3.1 ng/mL). In our study, children with MPIAP had slightly higher HBD2 levels: 23.70–603.2 ng/mL (median 58.35 ng/mL) vs. 23.1–97.8 ng/mL (median 47.50 ng/mL). Unfortunately, the HBD2 concentrations had low sensitivity (73%), so one in four children with MPIAP did not have their disease confirmed by this indicator.

## 5. Conclusions

The aim of our study was to investigate whether fECP and HBD2 are significantly elevated in children with MPIAP and whether a monthly milk-free diet reduces these markers. Faecal ECP and HBD2 can be used to monitor the resolution of colitis in infants with MPIAP treated with a milk diet, indicating a slower resolution of allergic inflammation than the resolution of allergic symptoms. Neither parameter is useful for the diagnosis of MPIAP. High concentrations of fECP can be helpful in diagnosing MPIAP (100% sensitivity), but the low specificity of the assay results in a risk of diagnosing MPIAP in one in six children who do not have the disease. The concentrations of HBD2 have low sensitivity, so one in four children with MPIAP will not have their disease confirmed using this indicator.

## Figures and Tables

**Table 1:** Characteristics of the study groups: children with milk protein-induced allergic proctocolitis (MPIAP_0_) and children from the control group.

Parameter	Study Group MPIAP_0_ n = 70	Control Group * n = 30	*p*
Age median (mo)	2 (1–3)	2 (1–3)	ns **
Males (n, %)	50 (71.4%)	24 (80%)	ns ***
Body mass median (centiles)	50 (10–97)	50 (10–97)	ns **
Height median (centiles)	75 (10–97)	75 (10–97)	ns **
Feeding at the time of diagnosis (n/%)			
Breast	64 (91.4%)	27 (90.0%)	ns ***
Breast + infant milk	4 (5.6%)	2 (6.7%)	
Only infant milk	2 (3.0%)	1 (3.3%)	
Allergies in family (n/%)	30 (42.8%)	13 (43.3%)	
Father	26 (37.1%)	9 (30.0%)	
Mother	25 (35.6%)	7 (23.3%)	ns ***
Siblings	32 (45.7%)	14 (46.7%)	
Symptoms (n/%)			
MPIAP ****	52 (74.2%)	-	
MPIAP + atopic	18 (25.8%)	-	
Dermatitis			
General symptoms (n/%)			
Anxiety	24 (34.2%)	5 (17.0%)	
Anaemia	6 (8.0%)	6 (20.0%)	<0.05 ***
Eosinophilia	16 (22.8%)	0 (0.0%)	
Milk-free diet during treatment (n/%)			
Breast	63 (90.0%)		
Breast + eHf *****	6 (8.6%)		
Only eHf *****	1 (1.4%)		

* Children with gastro-oesophageal reflux, in whom a relation of symptoms to milk allergy was excluded; ** Mann–Whitney U-test; *** Spearman’s rank correlation test; **** MPIAP—milk-protein-induced allergic proctocolitis; ***** eHf—extensively hydrolysed infant formula. ns non-significant.

**Table 2:** Faecal eosinophil cationic protein (fECP) and faecal β-defensin (HBD2) levels in children with milk-protein-induced allergic proctocolitis at diagnosis (MPIAP_0_), after 1 month of following a milk-free diet (MPIAP_1_), and in the control group.

Parameter	Control Group n = 30	Study Group MPIAP_0_ n = 70	Study Group MPIAP_1_ n = 70	*p* *
fECP (mg/L)				
median	0.22	2.36	0.47	<0.0001 **
range	0.11–1.47	1.03–5.46	0.14–2.58	
HBD2 (ng/L)				
median	3.10	58.35	9.10	
range	1.10–10.30	23.70–603.2	1.90–384.90	<0.0001 **

* Mann–Whitney U-test; ** MPIAP_0_/MPIAP_1_ and control group/MPIAP_0_ and control group/MPIAP_1_.

**Table 3:** Frequency of differential levels of faecal eosinophil cationic proteins (fECP) and faecal β-defensins (HBD2) in the study group at the time of diagnosis (MPIAP_0_) and 1 month after following a milk-free diet (MIAP_1_) compared with the control group.

Parameter	Control Group n = 30 n (%)	Study Group MPIAP_0_ n = 70 n (%)	Study Group MPIAP_1_ n = 70 n (%)	*p* *
fECP (mg/L)				
<1.005	25 (83.3%)		50 (87.7%)	<0.0001 **
≥1.005	5 (16.7%)	70 (100%)	7 (12.3%)	
HBD2 (ng/L)				
<19.35	30 (100%)	19 (27.1%)	49 (71.0%)	
≥19.35		51 (72.9%)	20 (28.9%)	<0.0001 **

* Spearman’s range correlation test; ** MPIAP_0_/MPIAP_1_ and control group/MPIAP_0_ and control group/MPIAP_1_.

## Data Availability

The results of the tests are included in the records of the outpatient clinics where the children were treated.
